# No Consistent Shift in Leaf Dry Mass per Area Across the Cretaceous—Paleogene Boundary

**DOI:** 10.3389/fpls.2022.894690

**Published:** 2022-06-16

**Authors:** Matthew J. Butrim, Dana L. Royer, Ian M. Miller, Marieke Dechesne, Nicole Neu-Yagle, Tyler R. Lyson, Kirk R. Johnson, Richard S. Barclay

**Affiliations:** ^1^Department of Earth and Environmental Sciences, Wesleyan University, Middletown, CT, United States; ^2^Department of Geology and Geophysics and Program in Ecology, University of Wyoming, Laramie, WY, United States; ^3^Department of Earth Sciences, Denver Museum of Nature and Science, Denver, CO, United States; ^4^U. S. Geological Survey, Denver, CO, United States; ^5^Department of Paleobiology, Smithsonian Institution, National Museum of Natural History, Washington, DC, United States

**Keywords:** Cretaceous—Paleogene boundary, leaf mass per area (LMA), mass extinction, paleobotanical analysis, Denver Basin, Williston Basin, leaf economic spectrum (LES), Castle Rock

## Abstract

The Chicxulub bolide impact has been linked to a mass extinction of plants at the Cretaceous—Paleogene boundary (KPB; ∼66 Ma), but how this extinction affected plant ecological strategies remains understudied. Previous work in the Williston Basin, North Dakota, indicates that plants pursuing strategies with a slow return-on-investment of nutrients abruptly vanished after the KPB, consistent with a hypothesis of selection against evergreen species during the globally cold and dark impact winter that followed the bolide impact. To test whether this was a widespread pattern we studied 1,303 fossil leaves from KPB-spanning sediments in the Denver Basin, Colorado. We used the relationship between petiole width and leaf mass to estimate leaf dry mass per area (LMA), a leaf functional trait negatively correlated with rate of return-on-investment. We found no evidence for a shift in this leaf-economic trait across the KPB: LMA remained consistent in both its median and overall distribution from approximately 67 to 65 Ma. However, we did find spatio-temporal patterns in LMA, where fossil localities with low LMA occurred more frequently near the western margin of the basin. These western margin localities are proximal to the Colorado Front Range of the Rocky Mountains, where an orographically driven high precipitation regime is thought to have developed during the early Paleocene. Among these western Denver Basin localities, LMA and estimated mean annual precipitation were inversely correlated, a pattern consistent with observations of both fossil and extant plants. In the Denver Basin, local environmental conditions over time appeared to play a larger role in determining viable leaf-economic strategies than any potential global signal associated with the Chicxulub bolide impact.

## Introduction

More than 50% of plant species in mid-continental North America went extinct at the Cretaceous—Paleogene boundary (KPB; ∼66 Ma; Wilf and Johnson, 2004; Nichols and Johnson, 2008; Lyson et al., 2019) in a mass extinction linked to the Chicxulub bolide impact (Alvarez et al., 1980; Vellekoop et al., 2014; Hull et al., 2020) and concurrent volcanism from the Deccan Traps (Courtillot et al., 1986; Schoene et al., 2019; Sprain et al., 2019). This event also caused the total extinction of non-avian dinosaurs (Le Loeuff, 2012) and led to significant ecological selection on insects and mammals (Labandeira et al., 2002; Grossnickle and Newham, 2016). The nature of ecological selection on plants is less understood. Extinction at the family level was negligible (Cascales-Miñana et al., 2018), and angiosperm dominated ecosystems were already well established by the Late Cretaceous (i.e., Johnson et al., 2003; Carvalho et al., 2021), raising the question of how the mass extinction caused functional changes in plant communities, if at all. One hypothesis suggests that an impact winter triggered by the bolide favored deciduous plants (Wolfe and Upchurch, 1986; Wolfe, 1987). Impact generated aerosols could have imposed a regime of global dim light and low atmospheric temperatures for months to years (Vellekoop et al., 2014), giving deciduous plants, naturally disposed to periods of dormancy, a competitive advantage over their evergreen counterparts.

The signal for a shift toward deciduous strategies is difficult to pick up in the fossil record. However, a proxy for estimating the functional trait leaf dry mass per area (LMA) is well suited for making inference about shifts in plant strategy, including between deciduous and evergreen leaf habit (Royer et al., 2007). This is because LMA estimates can be mapped onto the leaf economics spectrum (LES), a continuum of viable leaf strategies that reflects tradeoffs in leaf resource allocation (Wright et al., 2004). One end of the LES represents fast-return strategies, which are manifested as thin, flimsy leaves (low LMA) that trade a short leaf lifespan (often deciduous) for high rates of photosynthesis. The other end of the LES represents slow-return strategies, manifested as thick, tough, long-lived leaves (high LMA), that due to a low rate of photosynthesis only slowly make a return on their initial carbon investment. These trait relationships are robust across the angiosperm phylogeny and although particular clades can have distinctive trait values (Ackerly and Reich, 1999), including LMA (Cornwell et al., 2014), the same fundamental leaf economic tradeoffs still occur (Wright et al., 2004). Thus, even in a mass extinction scenario in which there was significant taxonomic turnover, shifts in trait values represent shifts in leaf economic strategies.

**FIGURE 1:**
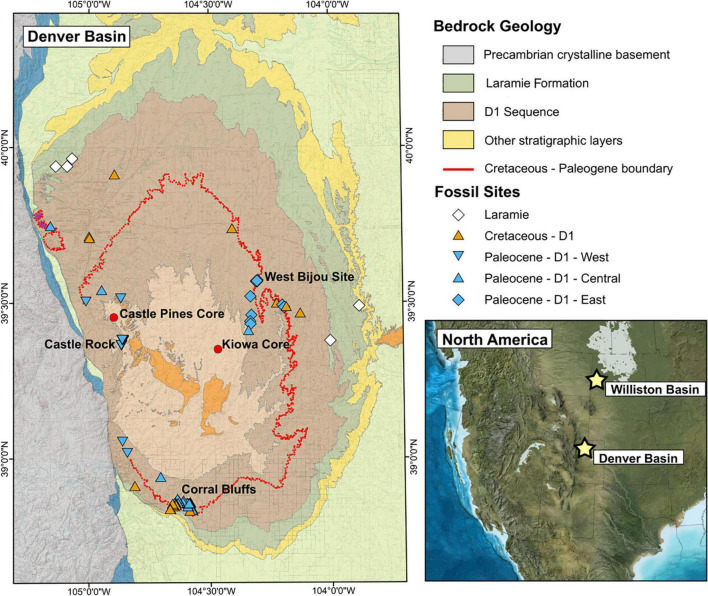
Bedrock geology of the Denver Basin (modified from Dechesne et al., 2011) and locations of megafloral fossil sites. The D1 Sequence in yellow and the Laramie Formation in pink are the two fossil-bearing sediments considered in this study. The inset map [Paleogeography of the North America Western Interior at the KPB, from Ron Blakely, Colorado Plateau Geosystems, Arizona, United States (http://cpgeosystems.com/paleomaps.html)] shows the relative position of the Williston and Denver Basins; the stars are in the general region of the fossil sites used from each basin.

Within this leaf economic framework, Blonder et al. (2014) studied Late Cretaceous and early Paleocene fossil plant localities from the Williston Basin, North Dakota (Figure 1), and found evidence for a loss of slow-return, high LMA species across the KPB; Wilson Deibel (2022) reports similar patterns further west in the basin. Both studies interpret their results to represent the increased survival of fast-return, likely deciduous, plants in response to an impact winter. This theory, reliant on a global-scale change in environment, suggests a global response. However, recent studies indicate that the angiosperm trait response to the mass-extinction event was geographically heterogeneous, highlighting different responses between South American floras and the North American Williston Basin flora (Stiles et al., 2020; Carvalho et al., 2021). Further, other hypotheses of selection across the KPB connect survival to traits not explicitly linked to the LES, such as non-recalcitrant seeds (Berry, 2020) and polyploidy (Fawcett et al., 2009; Moeglein et al., 2020; Wei et al., 2020).

In light of these recent findings, we test whether the shift toward fast-return strategies observed in the Williston Basin represents a common response to the global effects of the bolide impact. Following Blonder et al. (2014) we use LMA estimates to position fossil plant communities preserved in KPB-spanning sediments of the Denver Basin, Colorado (Figure 1) along the LES. The Denver Basin fossil record complements the Williston Basin well and offers new opportunities for interpreting LMA and the broader LES across the KPB. First, while the Late Cretaceous fossil record in the Williston Basin is better represented than the Paleocene record (Wilf and Johnson, 2004), the reverse is true in the Denver Basin (Johnson et al., 2003). Second, the Paleocene flora of the Williston Basin consists of a homogeneous low-diversity, basin-center flora within a broad, low-relief coastal plain (Johnson, 2002). In contrast, the synorogenic, KPB-spanning sediments in the Denver Basin, which were deposited adjacent to and during the uplift of the Colorado Front Range of the Rocky Mountains, contain a heterogeneous flora that includes both low-diversity basin-center localities and high-diversity mountain-proximal localities (Johnson et al., 2003). While the flora in the center of the Denver Basin provides a close taxonomic and ecological analog to the flora of the Williston Basin (i.e., the “Fort Union Flora,” see Nichols and Johnson, 2008), the mountain proximal flora provides an opportunity to evaluate the ecological strategies and responses of different plant assemblages, such as the early rainforest flora at Castle Rock (Johnson and Ellis, 2002).

To understand trans-KPB shifts in LMA in the Denver Basin, we evaluate four scenarios following Blonder et al. (2014). These are (1) directional shift—a shift in LMA space associated with a shift in environmental conditions; (2) convergence—a narrowing in LMA space associated with a loss of viability of ecological strategies; (3) divergence—a widening in LMA space associated with the appearance of new environmental conditions supporting newly viable strategies; and (4) a lack of environmental filtering—no significant changes in LMA expression because environmental changes are unrelated to LMA. In the Williston Basin, the loss of slow-return strategies was a combination of a directional shift and a convergence toward low LMA values (Blonder et al., 2014). If this represents a global response to the bolide impact, the Denver Basin flora should also exhibit a downward directional shift and a convergence in LMA space across the KPB, regardless of taxonomic and environmental differences between the basins. A KPB spanning subset of 39 localities in the Corral Bluffs study area of the Denver Basin (Figure 1) showed a moderate downward shift in both minimum and maximum LMA values but no significant shift in median LMA (Lyson et al., 2019), leaving it unresolved whether plant communities in the Denver Basin responded similarly to those in the Williston Basin. We present here a Denver Basin LMA analysis on a larger scale, spanning four million years across the KPB and ranging from the low-diversity basin center to the taxonomically and environmentally diverse floras of the Denver Basin’s mountain proximal western margin.

## Materials and Methods

### Data Sources

We estimated LMA on 1,303 fossil leaves from 95 localities in the Denver Basin (paleolatitude: ∼44–46° N; Van Hinsbergen et al., 2015), representing 551 species-site pairs. All measured leaves come from woody dicot angiosperms. Common taxa were described and many of the individual localities were listed by Johnson et al. (2003), with the remaining localities collected subsequently by the Denver Museum of Nature and Science (DMNS). The chronology of the Denver Basin is well constrained, with U-Pb dates for the KPB (66.021 ± 0.024 Ma) and the magnetochron boundaries C28 n through C30 n taken from the 688 m long Kiowa core near the center of the basin and along the West Bijou Creek escarpment (Clyde et al., 2016; Figure 1); individual localities within the basin can be constrained to ∼100 k.y. time bins or finer using radiometric dates and magnetochron boundaries (e.g., Clyde et al., 2016), combined with correlations between the Kiowa core, oil and water well logs throughout the basin, and surface outcrops (Raynolds et al., 2007; Dechesne et al., 2011). Many localities can be further constrained to ∼10 k.y. time bins by local sedimentation rates and a biostratigraphic framework derived from pollen, megaflora, and vertebrate fossils (Hicks et al., 2003; Johnson et al., 2003; Raynolds et al., 2007; Clyde et al., 2016; Lyson et al., 2019; see Supplementary Material for site age errors).

The measured fossils come from the Late Cretaceous Laramie Formation (∼69–68 Ma) and the KPB spanning D1 sequence of the Denver Basin Group (∼68–63.8 Ma; Johnson et al., 2003). The Laramie Formation consists of coal-bearing, backswamp floodplain facies associated with the withdrawal of the Western Interior Seaway (Raynolds and Johnson, 2003). The D1 sequence is an unconformity-bounded, synorogenic sedimentary package that contains the Arapahoe Formation (or Conglomerate; Raynolds, 2002), the Denver Formation, and the lower Dawson Formation (or Arkose). These interfingering formations, which were deposited adjacent to and during the uplift of the Colorado Front Range (Raynolds and Johnson, 2003), preserve floodplain facies deposited in environments that range from well- to poorly drained.

Cretaceous sediments of the D1 sequence are under-sampled compared to Paleocene sediments and contain a diverse and spatially heterogeneous flora (Johnson et al., 2003; Raynolds et al., 2007). As a result the characteristics of this flora are not fully understood, but it contains many species that appear to go extinct after the KPB, as well as many species also found in Cretaceous sediments of the Williston Basin (Johnson et al., 2003; Lyson et al., 2019).

Paleocene sediments of the D1 sequence are highly sampled and spatially heterogeneous. Much of the heterogeneity of the flora can be observed across a west-to-east profile shaped by proximity to the Colorado Front Range (Barclay et al., 2003; Ellis et al., 2003; Johnson et al., 2003). Based on this observation, Johnson et al. (2003) binned localities into three megafloral associations defined by geographic and stratigraphic occurrence (Figure 1): P-D1-West, P-D1-Central, and P-D1-East (“P” for Paleocene, “D1” for D1-sequence).

The P-D1-West floral association occurs in a narrow geographic band along the western margin of the basin close to the Front Range, and is generally found in high-energy floodplain systems associated with alluvial fans suggesting local topographic complexity. This association typically contains diverse floras with large leaves. A noteable site that falls within the P-D1-West association is Castle Rock (63.84 Ma; DMNH loc. 1,200, 2,689, 2,690, 2,698, 2,699, 2,716, 2,720, 2,723, 2,731, 2,733, 2,748, 2,763, 2,801, 2,802, 2,831, 2,966, 2,967, 2,968, 2,969, 2,994; Kowalczyk et al., 2018), an autochthonous leaf litter deposit which has been interpreted as an early Paleocene rainforest (Ellis and Johnson, 2013) very near to the Front Range (Ellis et al., 2003). The nearby Plum Creek Parkway (DMNH loc. 3,613, 3,618, 3,620) and Sick of Sycamores (DMNH loc. 2,339) localities (63.84 and ∼63.8 Ma, respectively) are not quite as diverse or as strongly diagnostic of rainforest physiognomies as Castle Rock, but are considered coeval floras found in different depositional settings on the floodplain (Ellis et al., 2003; Ellis and Johnson, 2013).

The P-D1-East floral association occurs in the eastern exposures of the basin, furthest from the Front Range. These localities are found in low-energy fluvial, lacustrine, or paludal systems indicating low paleo-relief, and the flora is typically low diversity (Barclay et al., 2003). The P-D1-East association shares a close taxonomic affinity with the Paleocene flora of the Williston Basin (Johnson et al., 2003) and is sometimes referred to as part of a widespread early Paleocene “Fort Union flora” (Nichols and Johnson, 2008), components of which can be found in similar swampy environments from the nearby Raton Basin (Wolfe and Upchurch, 1987) to the Ravenscrag Formation in Saskatchewan (West et al., 2021). Compared to the rest of the Paleocene D1, P-D1-East localities are sparse and do not represent as large of a temporal range, with most localities found nearer to the KPB (Johnson et al., 2003).

Last, the P-D1-Central floral association, while appearing geographically close to the western basin margin, is far enough from the Front Range that deposits are lower energy than the alluvial fan deposits characteristic of the P-D1-West floodplain systems. These P-D1-Central localities vary from low to high diversity and generally represent an intermediate flora with a smaller leaf size than is seen in the P-D1-West association but without the uniformly low diversity seen in the P-D1-East association. The Paleocene sites in the Corral Bluffs study area considered by Lyson et al. (2019; Figure 1) are part of the P-D1-Central association.

To evaluate changes across the KPB, we compared the Cretaceous D1 sequence floras (67.5–66.02 Ma; *n* = 17 localities) to the Paleocene D1 sequence floras that occur during the first one million years after the KPB (herein referred to as the “early Paleocene D1” flora; 66.02–65.02 Ma; *n* = 44). This categorization excludes the older Laramie Formation (*n* = 5) from the Cretaceous bin, and 29 younger Paleocene localities from between 64.75 and 63.8 Ma, including Castle Rock, from the Paleocene bin, resulting in a relatively continuous temporal sequence of measured fossils comparable in age range to fossils measured in the Williston Basin by Blonder et al. (2014).

### Morphotypes

The Denver Basin flora was initially classified with the morphotype method developed by Johnson (1989), using a separate set of morphotypes for five different study areas in the basin (Castle Rock—CR, Scotty’s Palm—SP, West Bijou Site—BC, Laramie Formation—LA, the remainder—JC; Johnson and Ellis, 2002; Barclay et al., 2003; Ellis et al., 2003; Johnson et al., 2003). The DMNS later partially integrated the five study areas into a single set of morphotypes covering the entire basin (Denver Basin—DB). The most common of these morphotypes were assigned to species and are typically found throughout the Denver Basin and sometimes in the Williston Basin as well (Johnson, 2002). The DMNS also made substantial collections at the Corral Bluffs study area in 2017 and 2018 (Lyson et al., 2019). These fossils have been assigned to a new morphotype series (Colorado Springs—CS) but are not integrated within the basin-wide DB system. Finally, a number of less-productive localities, along with many collected after Johnson et al. (2003), have not been integrated into either the DB or study area morphotype systems. Fossils from these localities have instead been assigned bin numbers, which are locality-specific classifications based on taxonomically important morphological characters (Ellis et al., 2009). Combined, the quality of taxonomy across these collections enables the analysis of morphospecies-site pairs (herein referred to as species-site pairs) with the understanding that bin numbers are not significant beyond their locality and that the morphotype series are only significant among the localities that the associated study area is comprised of.

### Leaf Mass per Area Measurements

**FIGURE 2:**
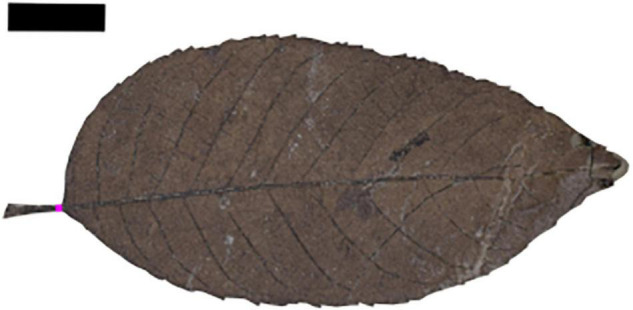
An example of leaf dry mass per area (LMA) estimation. Petiole width (0.07 cm) was measured at the pink line; leaf area (12.3 cm^2^) was estimated with a conservative reconstruction of the leaf apex. LMA = 60.5 g/m^2^. Morphotype DB396, EPI 53397. Baptist Road DMNH 2177 (65.7 Ma). Scale bar = 1 cm.

LMA estimates were made using the petiole width proxy of Royer et al. (2007), which depends on the mechanical relationship between the cross-sectional area of the petiole and leaf mass. We digitally photographed fossils at the DMNS using a Nikon D5300 digital camera with an af-s micro Nikkor 40 mm 1:2.8 g macrophotography lens, except for fossils from Castle Rock, which DMNS staff photographed using a Canon EOS Mark 5 d digital camera and 100 mm lens. We selected fossils with the petiole preserved widthwise at the base of the leaf blade and with enough of the leaf margin intact that we could confidently reconstruct the complete leaf area (Figure 2). Image manipulation and measurements were both done in Adobe Photoshop following Royer et al. (2007). Petiole width (PW) was measured with the ruler tool at the basal-most point of intersection between the petiole and the leaf blade. Leaf area (LA) was measured by tracing the margin of the leaf blade and petiole with the polygonal lasso tool, reconstructing any missing margin along the way, and then computing area with the measurement log toolbar (Figure 2). LMA was estimated with the linear regression derived by Royer et al. (2007) from a large set of leaves from 468 extant woody dicot species (Equation 1). 95% prediction intervals for each species site pair were derived following Sokal and Rohlf (2012) using the equation and coefficients provided by Royer et al. (2007; their Table 2). See Supplementary Material for all measurements.


(1) $$\log_{10}\text{LMA}=3.070+0.382\times \log_{10}\left(\frac{{\text{PW}}^{2}}{\text{LA}}\right)$$

### Climate Estimates

Estimates of mean annual temperature (MAT) and mean annual precipitation (MAP) for 77 and 11 localities respectively, come from previous reports (Ellis et al., 2003; Johnson et al., 2003; Lyson et al., 2019; see Supplementary Material). All MAT estimates used Wilf (1997) regression and all MAP estimates come from Johnson et al. (2003), preserving methodological continuity between locality climate estimates.

### Statistical Analysis

The significance of directional shifts in LMA was tested using the Mann-Whitney *U*-test, a non-parametric test of the null hypothesis that populations have the same median, and the Kolmogorov-Smirnov two-sample-test, a non-parametric test of the null hypothesis that two samples have an identical distribution (Sokal and Rohlf, 2012). The significance of convergence or divergence of LMA between sites was assessed using the Brown-Forsythe test, a non-parametric test of the null hypothesis that two samples have equal variance (Sokal and Rohlf, 2012).

### Comparison With Williston Basin

**FIGURE 3:**
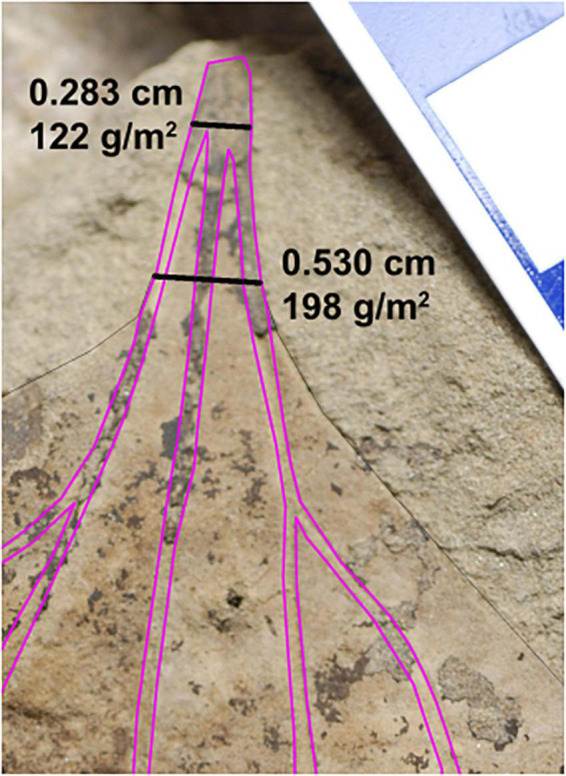
Representative revision from the Williston Basin. The two black lines are the revised petiole width measurement used in this study (top) and original measurement of Blonder et al. (2014) (bottom). Primary and secondary veins are traced with magenta lines. For each number pair, the top number is the petiole width and bottom is the estimated leaf dry mass per area. Morphotype HC162, *Marmarthia pearsonii*, DMNH 21687. Scale bar = 1 cm.

**FIGURE 4:**
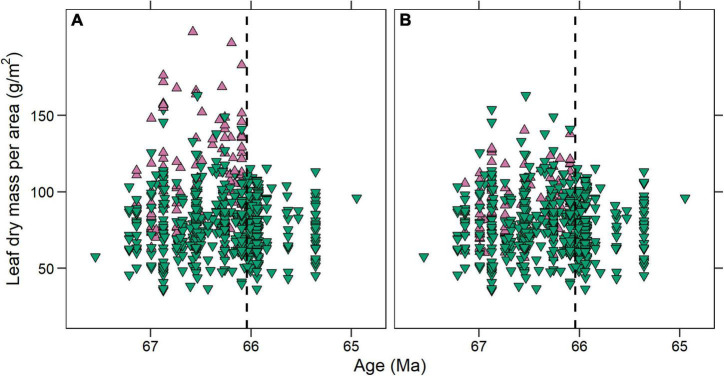
Individual leaf dry mass per area (LMA) estimates for the Williston Basin **(A)** before and **(B)** after revision. Upward facing purple triangles represent fossils that we measured again. The convergence in LMA space across the Cretaceous—Paleogene boundary (black dashed line) observed by Blonder et al. (2014) is still apparent after revision, although the loss of high-LMA leaves is less stark.

We also considered the 608 leaves representing 309 species-site pairs from the Williston Basin published by Blonder et al. (2014). Some morphotypes present in both basins have palmate venation with primary veins that converge beneath the point where the leaf margin intersects the outermost primary veins (Figure 3). Blonder et al. (2014) measured PW where the margin intersects these primaries; However, in many of these leaves, the primary veins are still separated from each other by laminar tissue at this point of intersection, causing an overestimation of PW and thus LMA. For this study, we measured PW directly below the point at which the primary veins fully converged. The difference in estimated LMA between these two methods was sometimes large, with the most extreme differences in excess of 100 g/m^2^ (Figure 4). Thus to better compare the two data sets, we remeasured all leaves from Williston Basin morphotypes with the described vein architecture, plus one morphotype with lamina along the length of the petiole that was previously included in the PW measurement (see Supplementary Material). We also removed morphotypes that Blonder et al. (2014) labeled herbaceous, because they require a different LMA regression, and because we solely focused on woody angiosperms in the Denver Basin. All comparisons to the Williston Basin use the revised LMA estimates.

In Blonder et al. (2014), Williston Basin sites were plotted by stratigraphic distance (m) from the KPB. In order to compare with the Denver Basin, we applied an age model based on an assumption of constant sedimentation rates between the KPB and magnetochron boundaries C30 n/C29 r and C29 r/C29 n (Hicks et al., 2002; Wilf et al., 2003). Boundary ages come from the GPTS (Gradstein et al., 2012). This results in a chronological range of fossils between ∼67.6 and 64.9 Ma.

## Results

### Basin-Wide Trends: Leaf Mass per Area at the Cretaceous-Paleogene Boundary

**FIGURE 5:**
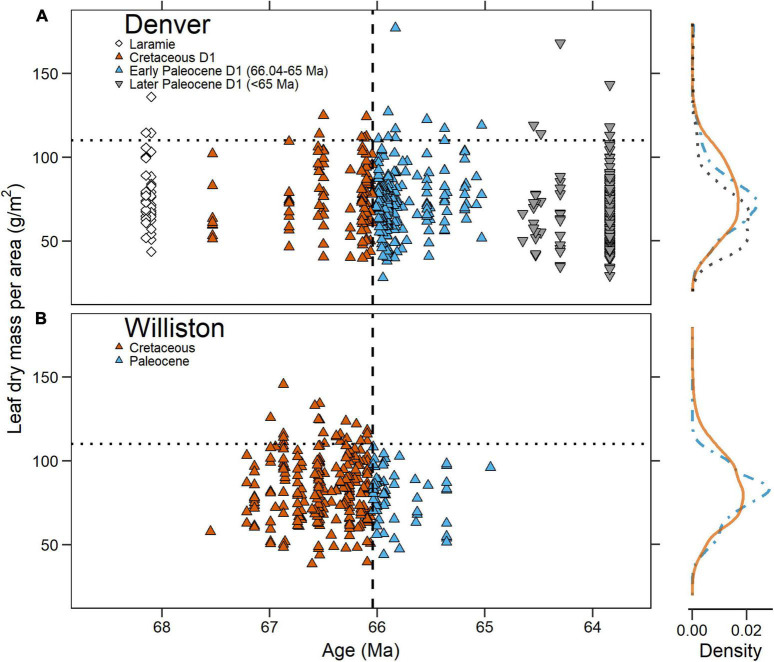
Mean leaf dry mass per area (LMA) of species-site pairs plotted against time in the **(A)** Denver Basin and **(B)** Williston Basin. In both, the vertical dashed line is the Cretaceous-Paleogene boundary and the horizontal dotted line is the 110 g/m^2^ cut-off for high LMA species observed in the Williston Basin. To the right are probability density functions for each basin. The solid orange line represents Cretaceous D1 species-site pairs in the Denver Basin and Cretaceous species-site pairs in the Williston. The blue dot-dash line represents early Paleocene D1 species-site pairs in the Denver Basin and Paleocene species-site pairs in the Williston. The black dotted line represents Later Paleocene D1 species-site pairs in the Denver Basin.

We found no statistically significant differences in LMA between species-site pairs in the Denver Basin’s Cretaceous D1 and early Paleocene D1 sediments (orange vs. blue symbols in Figure 5A). The median species-site pair LMA in Cretaceous D1 sediments (77.2 g/m^2^; *n* = 108) was not significantly different from the median species-site pair LMA in early Paleocene D1 sediments (72.4 g/m^2^; *n* = 196; *p* = 0.13). The distribution of species-site pairs in LMA did not significantly change across the KPB (*p* = 0.08), nor did the bounds of available LMA space as expressed by population variance (*p* = 0.15).

We estimated LMA of five KPB crossing morphotypes found in both Cretaceous and Paleocene sediments of the Denver Basin (Table 1). In the Cretaceous, the mean LMA of these boundary crossers ranged from 56.9 to 103.5 g/m^2^, suggesting that species occupying both the faster and slower-return ends of the LES survived the mass extinction. Similarly in the Paleocene the boundary crossers ranged from 48.4 to 104.2 g/m^2^, again spanning both ends of the spectrum. From the Cretaceous to the Paleocene, two of these morphotypes increased in LMA, while the other three decreased, but in all cases the 95% prediction intervals for the Cretaceous and Paleocene populations overlap suggesting that among the species that survived there was no unified leaf-economic response.

**TABLE 1:** Estimated mean leaf dry mass per area (LMA) of boundary crossing morphotypes found on both sides of the Cretaceous—Paleogene boundary (KPB).

KPB-crossing morphotypes	Cretaceous (g/m^2^)	Paleocene (g/m^2^)
*Platanites marginata*	$90.3\pm_{13.8}^{16.4}$	$72.5\pm_{7.2}^{8.0}$
*“Zizyphus” fibrillosus*	$103.5\pm_{29.3}^{41.0}$	$104.2\pm_{16.5}^{19.7}$
*“Ficus” planicostata*	$85.1\pm_{18.6}^{23.8}$	$71.3\pm_{10.7}^{12.5}$
DB 950	$73.9\pm_{16.2}^{20.7}$	$83.6\pm_{36.6}^{65.0}$
CSS 106	$56.9\pm_{21.4}^{34.2}$	$48.4\pm_{21.2}^{37.7}$

*Uncertainties are 95% prediction intervals.*

In the revised Williston Basin dataset (Figure 5B; see also section “Materials and Methods”), there was no significant shift (*p* = 0.19) in median LMA between the Cretaceous (83.0 g/m^2^; *n* = 237) and Paleocene (82.3 g/m^2^; *n* = 62) or in the distribution of LMA values (*p* = 0.12). Variance in LMA space however, decreased significantly across the KPB (*p* = 0.02); additionally, no Paleocene species-site pair had LMA greater than 110 g/m^2^, a level commonly exceeded in the Cretaceous (Figure 5B). Our analysis of revised measurements thus supports the original interpretation of Blonder et al. (2014) of a convergence in LMA space in the Paleocene.

In comparison with the Williston Basin, species-site pairs from the Denver Basin had significantly lower LMA in both the Cretaceous and Paleocene by median (respectively, *p* = 0.006; *p* = 0.005) and distribution (*p* = 0.04; *p* = 0.007). Variance in LMA space did not significantly differ between the two basins in either the Cretaceous (*p* = 0.54) or the Paleocene (*p* = 0.19). Despite the generally lower LMA of species-site pairs from the Denver Basin Paleocene, we observed nine with LMA exceeding the ceiling of 110 g/m^2^ observed in the Williston Basin Paleocene.

### Influence of Denver Basin Geography on Temporal Patterns

**FIGURE 6:**
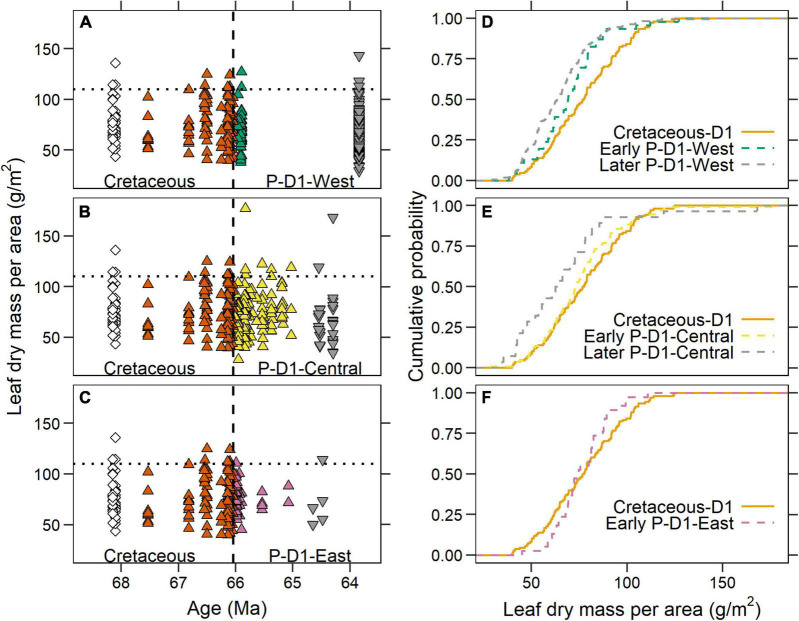
Leaf dry mass per area (LMA) of the three Paleocene D1 megafloral associations. **(A–C)** Comparisons of LMA in the Denver Basin between the Cretaceous and **(A)** P-D1-West; **(B)** P-D1-Central; and **(C)** P-D1-East associations. The Cretaceous species-site pairs are identical to Figure 5A and are identical in all three panels. In the Paleocene, upward facing triangles = early Paleocene D1 (66.04–65 Ma); downward facing triangles = later Paleocene D1 (< 65 Ma). The sum of Paleocene data across all three panels is identical to Figure 5A. **(D–F)** Cumulative distribution functions of LMA from different temporal groupings in the Denver Basin in **(D)** P-D1-West; **(E)** P-D1-Central; and **(F)** P-D1-East.

We observed different temporal responses in LMA among the three megafloral associations of the Paleocene D1 sequence (Figure 6). In the P-D1-West association, along the western margin of the basin, LMA shifted downwards after the KPB and stayed low throughout the first 2.2 m.y. of the Paleocene (Figures 6A,D). Compared to the Cretaceous D1, the early P-D1-West (*n* = 46 species-site pairs; green triangles in Figure 6A) had a significantly lower median LMA (69.1 vs. 77.2 g/m^2^; *p* = 0.03) and distribution (*p* = 0.03; Figure 6D), with no shift in variance (*p* = 0.12). Younger P-D1-West sites (gray triangles in Figure 6A)—Castle Rock, Sick of Sycamores, and Plum Creek Parkway (all ∼63.8 Ma)—were not significantly different from the early P-D1-West by median (63.3 g/m^2^; *n* = 167; *p* = 0.08), distribution (*p* = 0.19), or variance (*p* = 0.79).

The LMA of the early P-D1-Central association (*n* = 112) was not significantly different from the Cretaceous D1 by median (73.0 vs. 77.2 g/m^2^; *p* = 0.29), distribution (*p* = 0.31), or variance (*p* = 0.65; Figures 6B,E). However, we observed a later downwards shift in LMA more than a million years after the KPB (gray triangles in Figure 6B). Species from younger localities in P-D1-Central (*n* = 28) had a significantly lower median LMA (62.9 vs. 73.0 g/m^2^; *p* = 0.01) than the older early P-D1-Central assemblage, while distribution (*p* = 0.11) and variance (*p* = 0.44) were not significantly different.

The P-D1-East megafloral association showed no evidence of a downwards shift in LMA. The early P-D1-East (*n* = 38) did not differ from the Cretaceous D1 by median (74.1 vs. 77.2 g/m^2^; *p* = 0.86) or distribution (*p* = 0.33; Figures 6C,F). However, as in the Williston Basin, variance significantly decreased across the KPB (*p* = 0.007). Direct comparison between P-D1-East and the Williston Paleocene shows no significant difference in median (*p* = 0.18), distribution (*p* = 0.18), or variance (*p* = 0.61). Sparse sampling of species from younger sites in the P-D1-East association (*n* = 5; gray triangles in Figure 6C) makes it impossible to evaluate whether, like P-D1-Central, a downward shift in LMA occurred later.

### Correlation With Climate

**FIGURE 7:**
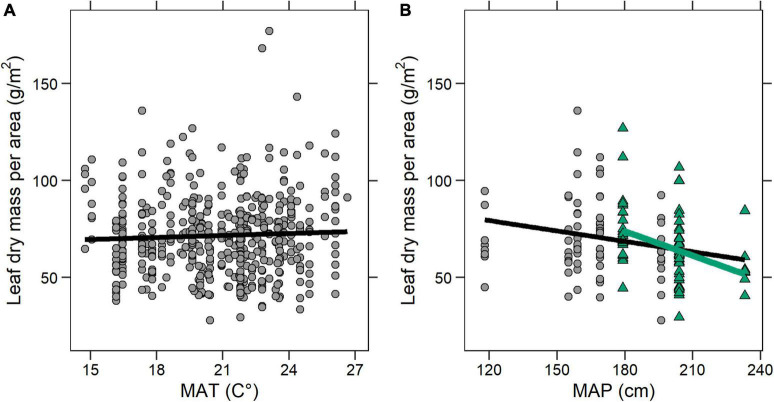
Correlations between LMA and climate. Each point is a species-site pair. **(A)** LMA and mean annual temperature (MAT). LMA = 63.25 + 0.40 × MAT (Adjusted *R*^2^ = 0.001; *p* = 0.20). **(B)** LMA and mean annual precipitation (MAP). Black line is the whole basin regression: LMA = 100.84 – 0.18 × MAP (Adjusted *R*^2^ = 0.05; *p* = 0.004). Green triangles are P-D-West species-site pairs, and the green line is the P-D1-West regression: LMA = 148.81–0.42 × MAP (Adjusted *R*^2^ = 0.14; *p* < 0.001).

We found a significant inverse correlation in the Denver Basin between MAP and LMA (*n* = 137 species-site pairs; adjusted *R*^2^ = 0.05; *p* = 0.003) and no correlation between MAT and LMA (*n* = 481; adjusted *R*^2^ = 0.01; *p* = 0.20; Figure 7). The MAP correlation was even stronger when restricted to the four P-D1-West sites with precipitation estimates (*n* = 70; adjusted *R*^2^ = 0.14; *p* < 0.001; green regression in Figure 7B).

## Discussion

We investigated over 1,000 leaves from nearly 100 localities temporally and spatially distributed throughout KPB-spanning Denver Basin sediments and found no evidence for a basin-wide change in plant ecological strategies. Our LMA estimates did not support a directional shift, convergence, or divergence across the KPB. Moreover, slow-return species with LMA greater than 110 g/m^2^, conspicuously absent in the Williston Basin post-KPB (Blonder et al., 2014; Figure 5B), were present throughout the Denver Basin record, including within less than 200 k.y. after the KPB (Figure 5A). Together, this suggests that the Williston Basin’s convergence in LMA after the KPB was not representative of a unified global, or even North American, response to the mass-extinction event.

Instead, our results indicate that shifts in LMA, even during this tumultuous time, were more reflective of smaller scale environmental factors. The P-D1-West megafloral association, located along the western basin margin next to the uplifting Front Range, experienced a directional shift toward lower LMA within the first 200 k.y. of the Paleocene and then persisted in that low LMA space through at least 63.8 Ma (Figures 6A,D). P-D1-Central, close to the basin margin but not directly adjacent to the uplifting Front Range, showed no significant change in LMA in relation to the KPB (Figures 6B,E; and see Lyson et al., 2019), but experienced a later directional shift toward lower LMA around 65–64 Ma. Finally, furthest from the basin margin, P-D1-East did not experience a directional shift in LMA at any point but did see a convergence in LMA within the first million years of the Paleocene (Figures 6C,F). This convergence in LMA without a corresponding directional shift closely matches what occurred in the Williston Basin during this same time span. The leaf-economic similarity is in keeping with a similarity in taxonomic composition and environmental setting (low-relief basin center).

We argue that the different temporal patterns observed in the western part of the basin (P-D1-West and P-D1-Central) could be a result of concurrent changes in precipitation. In the early Paleocene, surface relief generated during the uplift of the Colorado Front Range is thought to have initiated an orographic precipitation regime along the western margin of the basin (Sewall et al., 2000; Johnson et al., 2003; Sewall and Sloan, 2006). An inverse correlation between MAP and LMA is commonly observed in both extant (Wright et al., 2004, 2005) and fossil plants (Butrim and Royer, 2020). Thus, an increase in precipitation was likely one of the prevailing environmental factors most influencing plant strategies, and indeed we found a stronger inverse correlation between MAP and LMA among species-site pairs found in the P-D1-West association than elsewhere in the basin (Figure 7B). In the P-D1-Central and P-D1-East associations, we do not currently have enough MAP estimates to clearly link LMA to precipitation, but we hypothesize that a greater distance from the Front Range’s locally high paleo-relief and its associated orographic precipitation effects could have delayed the shift toward lower LMA until the uplift progressed further. In the P-D1-Central association, we see the first indication of a downward shift in LMA values about one million years after the KPB, around the same time that we see an increase in regional MAP in P-D1-West sites. While we do not have MAP estimates for the later P-D1-Central sites, it is possible that an intensification of orographic precipitation reached more Front Range distal environments, driving a downward shift. In the case of P-D1-East, the shift toward lower LMA values may have never occurred or occurred beyond the temporal range of available leaf fossils.

An exploration of individual sites bears out these same themes. Castle Rock (63.84 Ma; P-D1-West association) is notable for being an early example of a tropical rainforest (Johnson and Ellis, 2002; Johnson et al., 2003). Compared to most of the Denver Basin, Castle Rock had a low LMA (median = 64 g/m^2^) and a high MAP (204 cm), in-line with many present-day tropical rainforests (Royer et al., 2007; Poorter et al., 2009; Peppe et al., 2011). We found a similarly low LMA (median = 59 g/m^2^) at the older Baptist Road locality (∼66.5 Ma; P-D1-West; no MAP estimate), which is high in diversity but has never been classified as a rainforest. On the other hand, at Scotty’s Palm (∼66.5 Ma; P-D1-West), another high diversity locality found just after the KPB, LMA was higher (median = 80 g/m^2^) and MAP was lower (179 cm), in keeping with our basin-wide inverse correlation between MAP and LMA. Perhaps the difference in LMA between Baptist Road and Scotty’s Palm represents the beginnings of a transition to the later Castle Rock type rainforest, as changes in precipitation favored new leaf economic strategies. Future analyses incorporating taxonomy alongside these leaf-economic results should help reveal the nature and timing of this and other transitions by better describing the heterogeneity across localities and regions within the Denver Basin.

## Conclusion

The Denver Basin provides an opportunity to evaluate leaf economic responses to the end-Cretaceous mass extinction. In contrast to the Williston Basin, we found no basin-wide response in LMA across the KPB. Instead, changes in LMA were correlated with shifts in proximity to the Colorado Front Range and local precipitation. At P-D1-West sites, defined by high-diversity floras living adjacent to the Front Range with its orographic precipitation regime, we found evidence that MAP and LMA were negatively correlated. At the relatively Front Range distal P-D1-East sites, defined by low-diversity floras in the swampy basin center, we saw a narrowing in occupied LMA space similar to what occurred in the swampy depositional environments of the Williston Basin Paleocene. In the Denver Basin, localized environmental conditions, rather than the catastrophic mass extinction event, seem to have played the most important role in setting the limits of viable LMA space.

## Data Availability Statement

The original contributions presented in this study are included in the article/Supplementary Material, further inquiries can be directed to the corresponding author/s.

## Author Contributions

All authors listed have made a substantial, direct, and intellectual contribution to the work, and approved it for publication.

## Conflict of Interest

The authors declare that the research was conducted in the absence of any commercial or financial relationships that could be construed as a potential conflict of interest. The handling editor JS declared a past co-authorship with one of the authors DR.

## Publisher’s Note

All claims expressed in this article are solely those of the authors and do not necessarily represent those of their affiliated organizations, or those of the publisher, the editors and the reviewers. Any product that may be evaluated in this article, or claim that may be made by its manufacturer, is not guaranteed or endorsed by the publisher.

## References

[B1] Ackerly D. D. Reich P. B. (1999). Convergence and correlations among leaf size and function in seed plants: a comparative test using independent contrasts. *Am. J. Bot.* 86 1272–1281. 10.2307/2656775 10487815

[B2] Alvarez L. W. Alvarez W. Asaro F. Michel H. V. (1980). Extraterrestrial cause for the Cretaceous-Tertiary extinction. *Science* 208 1095–1108. 10.1126/science.208.4448.1095 17783054

[B3] Barclay R. S. Johnson K. R. Betterton W. J. Dilcher D. L. (2003). Stratigraphy and megaflora of a K-T boundary section in the eastern denver basin Colorado. *Rocky Mt. Geol.* 38 45–71. 10.2113/gsrocky.38.1.45 28159795

[B4] Berry K. (2020). Seed traits linked to differential survival of plants during the Cretaceous/Paleogene impact winter. *Acta Palaeobot.* 60 307–322. 10.35535/acpa-2020-0016

[B5] Blonder B. Royer D. L. Johnson K. R. Miller I. Enquist B. J. (2014). Plant ecological strategies shift across the Cretaceous–Paleogene boundary. *PLoS Biol.* 12:e1001949. 10.1371/journal.pbio.1001949 25225914 PMC4165584

[B6] Butrim M. J. Royer D. L. (2020). Leaf-economic strategies across the eocene-oligocene transition correlate with dry season precipitation and paleoelevation. *Am. J. Bot.* 107 1772–1785. 10.1002/ajb2.1580 33290590

[B7] Carvalho M. R. Jaramillo C. de la Parra F. Caballero-Rodríguez D. Herrera F. Wing S. (2021). Extinction at the end-Cretaceous and the origin of modern neotropical rainforests. *Science* 372 63–68. 10.1126/science.abf1969 33795451

[B8] Cascales-Miñana B. Servais T. Cleal C. J. Gerrienne P. Anderson J. (2018). Plants—the great survivors! Geol. *Today* 34 224–229. 10.1111/gto.12250

[B9] Clyde W. C. Ramezani J. Johnson K. R. Bowring S. A. Jones M. M. (2016). Direct high-precision U–Pb geochronology of the end-Cretaceous extinction and calibration of Paleocene astronomical timescales. *Earth Planet. Sci. Lett.* 452 272–280. 10.1016/j.epsl.2016.07.041

[B10] Cornwell W. K. Westoby M. Falster D. S. Fitzjohn R. G. O’Meara B. C. Pennell M. W. (2014). Functional distinctiveness of major plant lineages. *J. Ecol.* 102 345–356. 10.1111/1365-2745.12208

[B11] Courtillot V. Besse J. Vandamme D. Montigny R. Jaeger J. J. Cappetta H. (1986). Deccan flood basalts at the cretaceous/tertiary boundary? *Earth Planet. Sci. Lett.* 80 361–374. 10.1016/0012-821X(86)90118-4

[B12] Dechesne M. Raynolds R. G. H. Barkmann P. E. Johnson K. R. (2011). *Notes on the Denver Basin geologic Maps: Bedrock Geology, Structure, and Isopach Maps of the Upper Cretaceous to Paleogene Strata Between Greeley and Colorado Springs, Colorado, Denver, Colorado, Colorado Geological Survey map Series scale 1:250,000.* Denver, Co: Colorado Geological Survey.

[B13] Ellis B. Daly D. C. Hickey L. J. Johnson K. R. Mitchell J. D. Wilf P. (2009). *Manual of Leaf Architecture.* New York: Botanical Garden.

[B14] Ellis B. Johnson K. R. (2013). Comparison of leaf samples from mapped tropical and temperate forests: implications for interpretations of the diversity of fossil assemblages. *Palaios* 28 163–177. 10.2110/palo.2012.p12-073r 30628210

[B15] Ellis B. Johnson K. R. Dunn R. E. (2003). Evidence for an in situ early Paleocene rainforest from Castle Rock, Colorado. *Rocky Mt. Geol.* 38 73–100.

[B16] Fawcett J. A. Maere S. Van De Peer Y. (2009). Plants with double genomes might have had a better chance to survive the cretaceous-tertiary extinction event. *Proc. Natl. Acad. Sci. U.S.A.* 106 5737–5742. 10.1073/pnas.0900906106 19325131 PMC2667025

[B17] Gradstein F. M. Ogg J. G. Schmitz M. Ogg G. (eds) (2012). *The Geologic Time Scale.* Amsterdam: Elsevier.

[B18] Grossnickle D. M. Newham E. (2016). Therian mammals experience an ecomorphological radiation during the Late Cretaceous and selective extinction at the K–Pg boundary. *Proc. R. Soc. B Biol. Sci.* 283 1–8. 10.1098/rspb.2016.0256

[B19] Hicks J. F. Johnson K. R. Obradovich J. D. Miggins D. P. Tauxe L. (2003). Magnetostratigraphy of upper Cretaceous (Maastrichtian) to lower eocene strata of the denver basin. *Colorado. Rocky Mt. Geol.* 38 1–27. 10.2113/gsrocky.38.1.1 28159795

[B20] Hicks J. F. Johnson K. R. Obradovich J. D. Tauxe L. Clark D. (2002). Magnetostratigraphy and geochronology of the Hell Creek and basal Fort Union formations of southwestern North Dakota and a recalibration of the age of the Cretaceous-Tertiary boundary. *Spec. Pap. Geol. Soc. Am.* 361 35–55. 10.1130/0-8137-2361-2.35

[B21] Hull P. M. Bornemann A. Penman D. E. Henehan M. J. Norris R. D. Wilson P. A. (2020). On impact and volcanism across the cretaceous-paleogene boundary. *Science* 367 266–272. 10.1126/science.aay5055 31949074

[B22] Johnson K. R. (1989). *A High-Resolution Megafloral Biostratigraphy Spanning the Cretaceous-Tertiary Boundary in the Northern Great Plains.* New Haven, CT: Yale University.

[B23] Johnson K. R. (2002). Megaflora of the hell Creek and lower Fort Union Formations in the western Dakotas: vegetational response to climate change, the cretaceous-tertiary boundary event, and rapid marine transgression. *Spec. Pap. Geol. Soc. Am.* 361 329–391. 10.1130/0-8137-2361-2.329

[B24] Johnson K. R. Ellis B. (2002). A tropical rainforest in colorado 1.4 million years after the Cretaceous-Tertiary boundary. *Science* 296 2379–2383. 10.1126/science.1072102 12089439

[B25] Johnson K. R. Reynolds M. L. Werth K. W. Thomasson J. R. (2003). Overview of the late Cretaceous, early Paleocene, and early eocene megafloras of the denver basin, Colorado. *Rocky Mt. Geol* 38 101–120.

[B26] Kowalczyk J. B. Royer D. L. Miller I. M. Anderson C. W. Beerling D. J. Franks P. J. (2018). Multiple proxy estimates of atmospheric CO2 from an early Paleocene rainforest. *Paleoceanogr. Paleoclimatol.* 33 1427–1438. 10.1029/2018PA003356

[B27] Labandeira C. C. Johnson K. R. Wilf P. (2002). Impact of the terminal Cretaceous event on plant-insect associations. *Proc. Natl. Acad. Sci. U.S.A.* 99 2061–2066. 10.1073/pnas.042492999 11854501 PMC122319

[B28] Le Loeuff J. (2012). Paleobiogeography and biodiversity of Late Maastrichtian dinosaurs: how many dinosaur species went extinct at the Cretaceous-Tertiary boundary? *Bull. Soc. Geol. Fr.* 183 547–559. 10.2113/gssgfbull.183.6.547 28159795

[B29] Lyson T. R. Miller I. M. Bercovici A. D. Weissenburger K. Fuentes A. J. Clyde W. C. (2019). Exceptional continental record of biotic recovery after the Cretaceous-Paleogene mass extinction. *Science* 366 977–983. 10.1126/science.aay2268 31649141

[B30] Moeglein M. K. Chatelet D. S. Donoghue M. J. Edwards E. J. (2020). Evolutionary dynamics of genome size in a radiation of woody plants. *Am. J. Bot.* 107 1527–1541. 10.1002/ajb2.1544 33079383

[B31] Nichols D. J. Johnson K. R. (2008). *Plants and the KT Boundary.* Cambridge: Cambridge University Press.

[B32] Peppe D. J. Royer D. L. Cariglino B. Oliver S. Y. Newman S. Leight E. (2011). Sensitivity of leaf size and shape to climate: global patterns and paleoclimatic applications. *New Phytol.* 190 724–739. 10.1111/j.1469-8137.2010.03615.x 21294735

[B33] Poorter H. Niinemets Ü Poorter L. Wright I. J. Villar R. (2009). Causes and consequences of variation in leaf mass per area (LMA): a meta-analysis. *New Phytol* 182 565–588. 10.1111/j.1469-8137.2009.02830.x 19434804

[B34] Raynolds R. G. (2002). Upper Cretaceous and Tertiary stratigraphy of the Denver Basin, Colorado. *Rocky Mt. Geol.* 37 111–134.

[B35] Raynolds R. G. Johnson K. R. (2003). Synopsis of the stratigraphy and paleontology of the uppermost Cretaceous and lower Tertiary strata in the Denver Basin, Colorado. *Rocky Mt. Geol.* 38 171–181. 10.2113/gsrocky.38.1.171 28159795

[B36] Raynolds R. G. Johnson K. R. Ellis B. Dechesne M. Miller I. M. (2007). Earth history along Colorado’s Front Range: salvaging geologic data in the suburbs and sharing it with the citizens. *GSA Today* 17 4–10.

[B37] Royer D. L. Sack L. Wilf P. Lusk C. H. Jordan G. J. Niinemets Ü (2007). Fossil leaf economics quantified: calibration, eocene case study, and implications. *Paleobiology* 33 574–589. 10.1666/07001.1

[B38] Schoene B. Eddy M. P. Samperton K. M. Keller C. B. Keller G. Adatte T. (2019). U-Pb constraints on pulsed eruption of the Deccan traps across the end-Cretaceous mass extinction. *Science* 363 862–866. 10.1126/science.aau2422 30792300

[B39] Sewall J. O. Sloan L. C. (2006). Come a little bit closer: a high-resolution climate study of the early Paleogene Laramide foreland. *Geology* 34 81–84. 10.1130/G22177.1

[B40] Sewall J. O. Sloan L. C. Huber M. Wing S. (2000). Climate sensitivity to changes in land surface characteristics. *Glob. Planet. Change* 26 445–465. 10.1016/S0921-8181(00)00056-4

[B41] Sokal R. Rohlf F. (2012). *Biometry*, 4th Edn. New York, NY: WH Freeman and Company.

[B42] Sprain C. J. Renne P. R. Vanderkluysen L. Pande K. Self S. Mittal T. (2019). The eruptive tempo of deccan volcanism in relation to the cretaceous-paleogene boundary. *Science* 363 866–870. 10.1126/science.aav1446 30792301

[B43] Stiles E. Wilf P. Iglesias A. Gandolfo M. A. Cuneo N. R. (2020). Cretaceous-Paleogene plant extinction and recovery in Patagonia. *Paleobiology* 46 445–469. 10.1017/pab.2020.45

[B44] Van Hinsbergen D. J. J. De Groot L. V. Van Schaik S. J. Spakman W. Bijl P. K. Sluijs A. (2015). A paleolatitude calculator for paleoclimate studies. *PLoS One* 10:e0126946. 10.1371/journal.pone.0126946 26061262 PMC4462584

[B45] Vellekoop J. Sluijs A. Smit J. Schouten S. Weijers J. W. H. Sinninghe Damsté J. S. (2014). Rapid short-term cooling following the Chicxulub impact at the Cretaceous-Paleogene boundary. *Proc. Natl. Acad. Sci. U.S.A.* 111 7537–7541. 10.1073/pnas.1319253111 24821785 PMC4040585

[B46] Wei N. Du Z. Liston A. Ashman T. L. (2020). Genome duplication effects on functional traits and fitness are genetic context and species dependent: studies of synthetic polyploid Fragaria. *Am. J. Bot.* 107 262–272. 10.1002/ajb2.1377 31732972

[B47] West C. K. Reichgelt T. Basinger J. F. (2021). The Ravenscrag Butte flora: paleoclimate and paleoecology of an early Paleocene (Danian) warm-temperate deciduous forest near the vanishing inland Cannonball Seaway. *Palaeogeogr. Palaeoclimatol. Palaeoecol.* 576:110488. 10.1016/j.palaeo.2021.110488

[B48] Wilf P. (1997). When are leaves good thermometers? A new case for leaf margin analysis. *Paleobiology* 23 373–390.

[B49] Wilf P. Johnson K. R. (2004). Land plant extinction at the end of the Cretaceous: a quantitative analysis of the North Dakota megafloral record. *Paleobiology* 30 347–368. 10.1666/0094-83732004030<0347:lpeate<2.0.co;2

[B50] Wilf P. Johnson K. R. Huber B. T. (2003). Correlated terrestrial and marine evidence for global climate changes before mass extinction at the Cretaceous-Paleogene boundary. *Proc. Natl. Acad. Sci. U.S.A.* 100 599–604. 10.1073/pnas.0234701100 12524455 PMC141042

[B51] Wilson Deibel P. K. (2022). *Vegetation and Environmental Changes Across the Cretaceous-Paleogene (K-Pg) Boundary in Northeastern Montana.* Seattle, WA: University of Washington.

[B52] Wolfe J. A. (1987). Late Cretaceous-Cenozoic history of deciduousness and the terminal Cretaceous event. *Paleobiology* 13 215–226. 10.1017/S0094837300008769

[B53] Wolfe J. A. Upchurch G. R. (1986). Vegetation, climatic and floral changes at the Cretaceous-Tertiary boundary. *Nature* 324 148–152. 10.1038/324148a0

[B54] Wolfe J. A. Upchurch G. R. (1987). Leaf assemblages across the Cretaceous-Tertiary boundary in the Raton Basin, New Mexico and Colorado. *Proc. Natl. Acad. Sci.* 84 5096–5100. 10.1073/pnas.84.15.5096 16593859 PMC298800

[B55] Wright I. J. Reich P. B. Cornelissen J. H. C. Falster D. S. Groom P. K. Hikosaka K. (2005). Modulation of leaf economic traits and trait relationships by climate. *Glob. Ecol. Biogeogr.* 14 411–421. 10.1111/j.1466-822x.2005.00172.x

[B56] Wright I. J. Reich P. B. Westoby M. Ackerly D. D. Baruch Z. Bongers F. (2004). The worldwide leaf economics spectrum. *Nature* 428 821–827. 10.1038/nature02403 15103368

